# The critical elements of digital health in diabetes and cardiometabolic care

**DOI:** 10.3389/fendo.2024.1469471

**Published:** 2024-09-16

**Authors:** Mansur Shomali, Pablo Mora, Grazia Aleppo, Malinda Peeples, Abhimanyu Kumbara, Janice MacLeod, Anand Iyer

**Affiliations:** ^1^ Welldoc, Columbia, MD, United States; ^2^ University of Texas Southwestern Medical Center, Dallas, TX, United States; ^3^ Feinberg School of Medicine, Northwestern University, Chicago, IL, United States; ^4^ Janice MacLeod Consulting, Glen Burnie, MD, United States

**Keywords:** diabetes, digital health, artificial intelligence, connected health, digital therapeutics, self-management, quintuple aim

## Abstract

Digital innovations provide novel opportunities to individualize a person’s care to best match their lifestyle needs and circumstances and to support them as they live their daily lives with diabetes. These innovations also serve to provide actionable data and insights for the care team giving them a “Webb telescope-like” view into their individual self-management journey, allowing them to see what cannot be seen during infrequent and limited office visits, thereby facilitating collaboration and communication to optimize the care plan on a timely basis. Technology advances are enabling diabetes care to transition from episodic, synchronous, primarily in-person care to include synchronous virtual care options and to continuous, on-demand, data-informed, asynchronous digital care better matching the demands of living with a relentless 24/7 chronic condition. In this paper we will discuss the critical elements and considerations in designing and implementing successful diabetes digital health tools in clinical practice.

## 1 Introduction

The rising global burden of diabetes and related cardiometabolic conditions (1, 2), combined with a worsening global shortage of healthcare professionals (3), necessitates new approaches to expand access to care, lessen the burden on individuals living with these conditions, improve efficiencies and reduce unsustainable medical costs. Diabetes is a condition that heavily relies on self-management on the part of individuals, requiring them to perform and track multiple daily tasks as outlined in the Association of Diabetes Care and Education Specialists’ self-care behaviors framework (ADCES7 Self-Care Behaviors^®^). These behaviors include the management of medications, glucose, activity, diet, coping, risk, and problem solving (4). As such, diabetes self-management can be complex and challenging; only about half of individuals diagnosed with diabetes meet the American Diabetes Association (ADA) treatment targets (5, 6). Each person may have unique physical characteristics, emotional concerns and environmental circumstances that could impact their ability and sometimes willingness to self-manage their condition (7). Digital innovations provide novel opportunities to individualize a person’s care to best match their lifestyle needs and circumstances and to support them as they live their daily lives with diabetes. These innovations also serve to provide actionable data and insights for the care team giving them a view into their individual self-management journey, allowing them to see what otherwise cannot be seen during infrequent and limited office visits, thereby facilitating collaboration and communication to optimize the care plan on a timely basis.

Digital advancements enable going beyond the current and traditional glycemic-centric approach to diabetes care to address an expanded set of risk factors (hypertension, hyperlipidemia, obesity, sleep, cardiac function), which are collectively referred to as cardiometabolic health management. Digital health capabilities lend themselves well to the free-living, behavioral aspects of self-care as many of the challenges of managing complex cardiometabolic conditions, such as diabetes and obesity occur in daily life, not when the individual is with their clinical team. Having a common, ubiquitous personal device on hand, which also doubles as a coach for helping manage diabetes, and to track, measure, advise, connect to others, and to nudge toward better health behaviors just-in-time and right when needed, can better support individuals between healthcare encounters.

## 2 What is digital health?

The Food and Drug Administration (8) defines the broad scope of digital health including categories such as mobile health, health information technology, wearable devices, telehealth and telemedicine, and personalized medicine. This includes mobile medical apps, clinical decision support software, and artificial intelligence (AI). Digital technology has led to a revolution in health care and has the potential to help clinicians more accurately diagnose and treat disease and improve the health care experience for the individual. Digital health technologies use mobile computing platforms, connectivity, software, and health care sensors spanning a wide range of uses, from general wellness applications to technologies intended for use as a medical product, in a medical product, as companion diagnostics, or as an adjunct to other medical products [devices, drugs, and biologics] (8). A digital therapeutic is health software intended to treat or alleviate a disease, disorder, condition, or injury by generating and delivering a medical intervention that has a demonstrable positive therapeutic impact on a patient’s health. Digital therapeutics meet the following criteria: Incorporate design, manufacturing, and quality best practices; engage end users in product development and usability processes; incorporate patient privacy and security protections; apply product deployment, management, and maintenance best practices; publish trial results inclusive of clinically meaningful outcomes in peer-reviewed journals; are reviewed and cleared or certified by regulatory bodies as required to support product claims of risk, efficacy, and intended use; make claims appropriate to clinical evaluation and regulatory status; collect, analyze, and apply real-world evidence and/or product performance data (9).

## 3 How is healthcare evolving with connected technologies and digital health?

The availability of a growing variety of connected technologies such as continuous glucose monitoring systems (CGMs), blood pressure monitors, smart scales, digital stethoscopes, automated insulin delivery, connected insulin pens, and other digital health devices including consumer-facing fitness trackers are enabling individuals with diabetes to measure and self-manage their health directly using the data collected as they live their daily lives (10). Data aggregators such as Tidepool (Palo Alto, California, USA) and Glooko (Palo Alto, California, USA) can be used to integrate data from various sources for people with diabetes and their care team. The goal of AI-powered digital healthcare is to collect and analyze the various data generated by an individual person and to support them by providing software-driven personalized coaching and insights, but to implement this “n=1 solution” at scale and across diverse populations and geographies.

**Figure 1:**
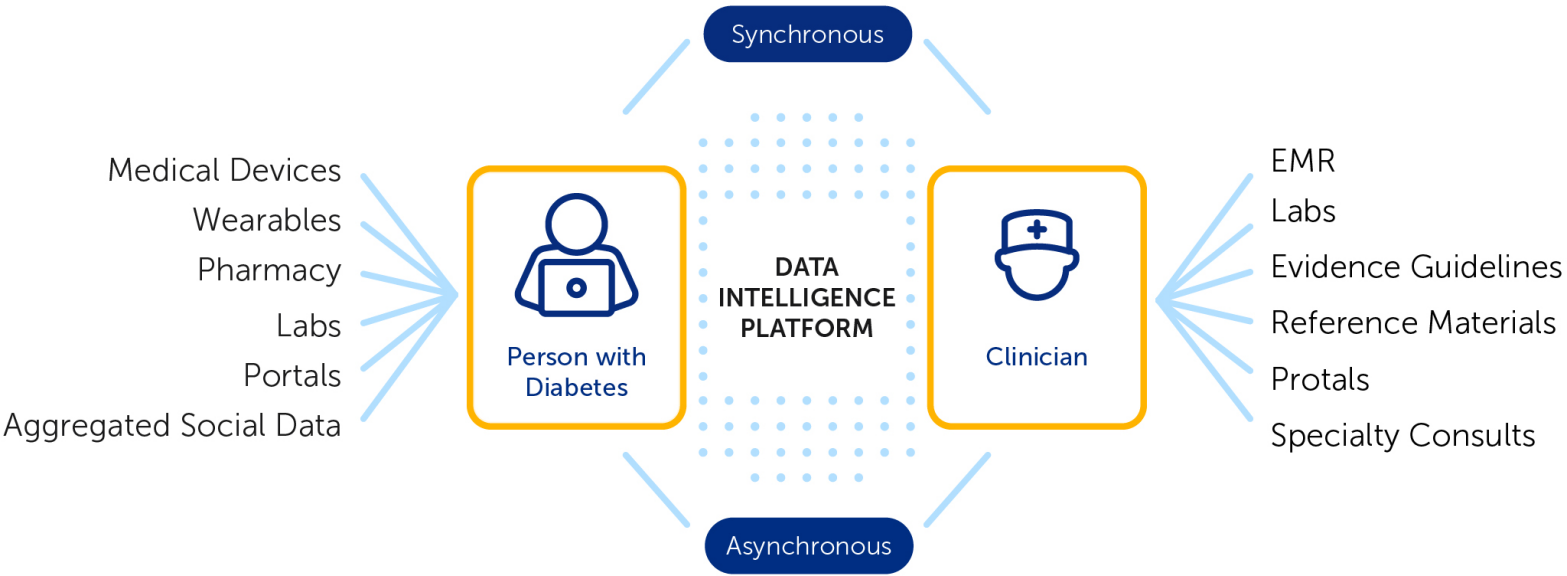
AI-Powered Digital Health Technology.

A vital element of success with digital health tools is strengthening the individual digital health user connection to their clinical care team. Digital health tools generate population- and person-level data, enabling healthcare teams to remotely monitor the health of populations more efficiently (11). With the increasing use of text messaging, regulated digital health apps, patient portals, digital social networks and video telehealth, it has become easier for clinicians to interact remotely with patients (12). A population health management approach that informs more effective and timely touchpoints with the clinical team when needed has the potential to help address health disparities and improve outcomes for individuals and populations (13). AI-powered digital health technology can be harnessed to realize practice efficiencies and to improve access to and effectiveness of care (7, 10, 11) as illustrated in **Figure 1**.

A public-private-industry partnership in the frontier state of Montana illustrated this concept. As part of a collaborative agreement between the Montana Diabetes Program (MDP) and the Centers for Disease Control (CDC), the MDP sought to identify opportunities to expand access and participation in diabetes self-management and education services (DSMES) across the large, mostly rural state. The goal was to explore and test innovative digital tools to eliminate barriers to participation and retention in underutilized DSMES services. The Montana Diabetes Digital Health Learning Network was formed as a collaborative project among the Montana public health department, Montana Coordinating Body of the ADCES, and Welldoc, a cardiometabolic digital health company. Eleven diabetes care and education specialists (DCESs) enrolled 198 patients with T1D or T2D in using the BlueStar^®^ digital health solution per its associated indications for use. Engaged participants achieved improvements in glycemia, blood pressure and weight (14). The DCESs were able to extend their DSMES services remotely including automated digital self-management support and iteratively develop best practices for remote physiological monitoring and population management.

An umbrella review sought to identify the active ingredients and mechanisms of action of technology enabled solutions for diabetes care (15). Here we list the mechanisms of action for digital health and digital therapeutic solutions:

- Lifestyle or behavioral modification, particularly for diabetes and obesity but also with associated comorbidities in mind (hypertension and hyperlipidemia) and/or smoking cessation and physical activity recommendations.
- Therapeutics, both insulin-based therapies (basal and basal/bolus) and non-insulin agents
- Biometrics, either to monitor vital information and to track progress with an intervention (glucose, blood pressure, heart rate, weight, etc.)
- Safety, addressing hypoglycemia, side effects with incretin therapies, sick day management, diabetes ketoacidosis, pancreatitis, etc.

Diabetes care is transitioning from episodic, synchronous, primarily in-person care to include synchronous virtual care options and to continuous, on-demand, data-informed, asynchronous digital care better matching the demands of living with a relentless 24/7 chronic condition (10, 12). Many digital health solutions for diabetes provide live coaching on-demand for example, MySugr (Vienna, Austria) integrated with the Roche Diabetes Care Platform, Livongo by Teladoc Health (Purchase, New York, USA), Virta Health (San Francisco, California, USA) and Verily Onduo (San Francisco, California, USA). Others, such as the Welldoc application (Columbia, Maryland, USA) don’t rely on live human-based coaching but rather, provide automated, scalable AI-powered coaching in real-time for individuals with integrated population-level and person-level data available to the care team, thus enabling remote monitoring and establishing criteria for clinical team intervention as needed. It is important to establish clear roles and responsibilities for clinical team members regarding who will receive, review and respond to population and person level data as digital-powered care models evolve.

Numerous challenges will need to be addressed as digital health advances. Expanding access to technology will require public-private partnerships to address the digital divide and technology literacy limitations. Additional clinical and economic evidence is needed to continue to demonstrate the value of digital health. Making digital health a mainstream aspect of care delivery requires significant buy-in from care teams and other healthcare stakeholders. To help build the case, leaders must outline digital health’s benefits for all stakeholders, describe how to attain those benefits, and back up the claims with clinical and real-world evidence.

Workflow integration best practices are essential. Digital solutions must be seamless components of care delivery, and solutions should be incorporated into the workflow, so they don’t create additional work or cause inefficiencies. The goal is to integrate digital health into mainstream care delivery, so it becomes as routine as in-person visits. Person-generated health data should be incorporated into the data view that care managers have access to so that they can address gaps in care, care transitions, and readmission and gain deeper insights into the health of their population. Engaging and activating individuals in their own health journeys demands a coordinated effort across extended-care teams, all of them working with the same data and understanding of a person’s health status. Through digital health, people become more empowered to self-manage their health and wellness when they have more meaningful interactions with their own personal care team. For individuals reluctant to engage, healthcare organizations should establish and implement support programs that walk people through their initial experiences, offer clear assurances that data is adequately encrypted, and make clear that they own their own data. Optimal engagement starts with clear support from a person’s own care team—through either a provider including a digital solution as part of the treatment plan or through a strong recommendation from a health plan care manager, a coach, a technology champion within the practice or a physician. Providing initial and ongoing support in use of the digital tool configured to the user’s care plan is essential as discussed later.

Building AI-enabled digital health capabilities is complex, requiring diligence in operationalizing extensive and diverse data sets, clinical evidence, data governance, interoperability and the use of a robust data intelligence platform that ensures privacy, security and scalable application in real-world settings. Good data governance ensures the mitigation of potential harm and helps to build and maintain public trust. The General Data Protection Regulation (GDPR) was instituted in 2018 providing comprehensive and robust data protection regulation across the globe. While GDPR addresses general personal data, the Health Insurance Portability and Accountability Act (HIPAA) enacted in the United States in 1996, focuses specifically on protecting medical information. HIPAA sets standards for the protection of health information and requires healthcare providers, insurers, and their business associates to implement strict data security measures. HIPAA compliance is critical for healthcare organizations to ensure the confidentiality, integrity, and availability of protected health information (PHI).

Additionally, payment models will need to be redesigned to accommodate digital health. This will require digital health companies to quantify how digital solutions can lower costs and improve efficacy compared with other modes of care delivery or in combination with other modalities.

## 4 Does digital health require regulatory oversight and need to be prescribed?

As noted above, the FDA acknowledges the wide span of digital health uses from general wellness applications to technologies intended for use as, in or as an adjunct to a medical product or therapy (8). Diabetes digital health solutions offering basal insulin dose titration such as the Tempo system by Lilly (Indianapolis, Indiana, USA) require regulation and are prescribed. Digital health applications such as Dreamed insulin decision support system (Petah Tikva, Israel) are provider facing and require both regulatory oversight and a prescription. In some cases digital health tools include functions requiring regulation and prescriptions as well as general health and wellness functions not requiring regulation or prescriptions. For example, in smart insulin pens, such as the InPen system by Medtronic (Northridge, CA, USA) and the Tempo system by Lilly (Indianapolis, Indiana, USA), the insulin dose calculator is regulated while dose reminders and dose tracking functions are not regulated. Other digital health solutions, such as the Welldoc application for cardiometabolic conditions (Columbia, Maryland, USA) include specific features that require a prescription and regulatory oversight, such as the rapid and basal insulin dose titration functionality, while other features, such as food and activity tracking and coaching, do not require either a prescription or regulatory review. Both are critical for success.

The product code used by the FDA for CGM-informed insulin bolus calculators has additional FDA requirements such as data simulation and clinical safety trials. A study to demonstrate the safety of a CGM-informed insulin bolus calculator that applies trend arrow and exercise adjustments to bolus insulin dose recommendations and provides real-time coaching found that use of the CGM-informed insulin bolus calculator by individuals with diabetes was associated with significant improvement in time-in-range (TIR; 70-180 mg/dL) without an increase in hypoglycemia or diabetes distress (16). Recently over-the-counter CGMs have been approved by the FDA offering sensor platforms with a limited set of functions to serve a broader scope of the population.

Real-world data analysis from connected insulin injection technologies is shining the spotlight on injection therapy revealing significant gaps and opportunities to improve care in this population (17). Visibility to dose data enables health care professionals to have a more complete picture of the pharmaco-adherence of insulin dosing informing the need to either address barriers to taking insulin as prescribed or making adjustments in the insulin plan (18). The paper logbook with its inaccuracies and omissions is being replaced with automated dose and glucose capture and data sharing. Smart insulin pens that objectively track active insulin are making it possible to calculate and deliver more frequent correction doses to safely improve glycemia without compromising time-below-range, thus changing the treatment paradigm for insulin injection therapy (17). A retrospective cohort analysis was conducted in 5,135 smart insulin pens users with either T1D or T2D, pediatric and adult, with their smart insulin pen paired with a personal CGM. Researchers used a rate-of-change detection methodology to identify meal events and timeliness of insulin doses. A dosing frequency of three or more times per day (including correction-only doses) and a missed dose frequency of less than 20% were associated with improved glycemia.

For further examples of digital health in diabetes care, readers are referred to an annual review of the literature highlighting key papers written in the prior year in which digital health technologies were used to provide digitally driven care for people with diabetes or prediabetes (10). For a thorough review of commercially available digital health solutions for diabetes the reader is referred to Doyle-Delgado and Chamberlain, 2020 (19).

## 5 What are the essential elements of digital health in diabetes care?

Successful diabetes digital health solutions keep the person in the center engaging and empowering them in their own care. Essential elements include:

### 5.1 Going beyond A1C

Digital health is not a substitute but rather, an amplifier of current standards, supporting various behaviors beyond glucose (the ADCES7 self-care behaviors (4) and bringing this data to life to drive “whole person care.” In addition, there is an opportunity to go beyond the usual ABCs of diabetes (A1C, blood pressure, and cholesterol [lipids]) by monitoring activity, sleep, weight, psycho-social wellness and social determinants of health such as food security, diabetes distress, and depression. Recognizing that many live with more than one chronic metabolic condition, digital health can bring an integrated holistic approach to self-management and care. For example, Welldoc reported results from a real-world study examining the use of a digital health tool with AI-coaching for people with diabetes enrolled in a virtual diabetes program. Of the 71 participants, 77% had significant weight loss (average weight loss was 4.8% of body weight). In addition, the participants enjoyed an average systolic blood pressure reduction of 7 mmHg (p=.01) (20).

### 5.2 Regulatory oversight

Higher risk digital health features must embrace evidence-based guidelines and deliver tailored interventions to people living with diabetes in a safe and effective manner. Thus, regulatory oversight is critical, to ensure a discipline and rigor for safety and efficacy, and also the implementation and management of good manufacturing processes, that support scalability, repeatability and traceability of quality and cybersecurity standards throughout the digital health lifecycle. Regulated digital health solutions can be combined and work together with general health and wellness digital health tools to provide a comprehensive solution. The digital health field continues to evolve as new technology capabilities and regulations are considered. As the digital health industry continues to develop, flexibility, safety & risk and real-world feasibility will need to be assessed in determining the appropriate regulatory oversight and integration into care.

### 5.3 Device integration and addressing the digital divide and technology literacy

Smartly engineered digital health takes advantage of hardware that people with diabetes already have and use. Digital health solutions engineered to work with existing hardware maximizes the return from dollars already invested and avoids the need to purchase and train individuals on new devices. The digital divide has been identified as a social determinant of health and will need to be addressed to expand digital health access and adoption (21). Well-designed digital health architectures can help address the digital divide and allow for use offline with episodic connectivity. Off-line use is of particular importance to ensure compliance with patient safety best practices. When setting up a new digital health tool account, providing SMS versus email account confirmation increases access to those without an email account. Manufacturers of CGMs should continue to offer the option of a standalone receiver versus needing to rely on a smartphone to expand access. Having a designated digital champion or technology navigator in the clinic can further help build efficiencies in technology onboarding and data accessibility. It will also be critical to build health technology literacy and confidence among those living with chronic conditions (13). In addition, makers of digital technologies need to consider how to meet the needs of those with low literacy or numeracy and sensory or motor impairment. As the population ages, there is growing evidence demonstrating the efficacy of diabetes technology for adults in their sixties, though data is limited for the oldest populations (22). ADA standards of care are now recommending AID systems (for those with T1D) and other advanced insulin delivery devices such as connected pens for older adults to reduce the risk of hypoglycemia (23).

### 5.4 Connected ecosystems and clinical integration for practice efficiency

Digital health gives visibility to population health data enabling remote monitoring capability, enabling true person-centered care for populations at scale (one-to-one to one-to-many). Digital health enables identifying who in the population needs a human touchpoint or nudge and then uses the person-level data to inform the interaction. Digital health provides the capability to segment the baseline population by various vectors enabling a more effective bottom-up vs. top-down approach to population health (i.e., people are heterogenous, and aggregated data is more meaningful at a population level). Digital health, if properly deployed, brings individuals and clinical teams together; visibility to data gives a window into the individual’s self-management journey since the last encounter and facilitates meaningful conversations and care plan adjustments.

### 5.5 Increasing role of artificial intelligence

Artificial intelligence, defined as software that learns, is revolutionizing diabetes care by providing advanced tools for monitoring, personalized treatment, and early detection of complications. By combining AI with human expertise, diabetes management can be transformed and improve outcomes for people living with diabetes. AI allows translation of general guidelines into consistent personalized delivery at scale with healthcare team oversight. It drives the translation of data to information, to knowledge, to action and ultimately, to outcomes. Simply put, right person, right place, right time, right instruction. AI democratizes access to diabetes management best practices in a manner that fits into a person’s “life-flow” and a healthcare provider’s workflow. AI capabilities are continuing to advance and allow us to pivot from a generalized (one-size-fits-all) approach to care and from technology guided by rules or algorithms to provide precise, holistic therapy recommendations that address the needs of each individual. Generative AI will be able to analyze a patient’s specific health data to provide tailored prevention recommendations, interpreting their data in the context of each person’s health history and treatment plans. The current evidence-based medicine represents the tip of the iceberg providing barely enough shallow evidence to care for a generic patient. To achieve the next-generation of deep evidence-based medicine, it will be necessary to gather and analyze all available data (natural history data, genomic, all published clinical studies, real-world data, and amassed data from Internet of medical things) (24).

AI is already being used in diabetes-cardometabolic care. CGMs use AI algorithms to analyze data from the devices to provide real-time insights into blood glucose trends and patterns. AI can predict high and low blood glucose events before they occur, allowing for timely interventions) (16). AI is being used in optimizing insulin pump therapy in the form of Automated Insulin Delivery (AID). AI is used to automatically adjust insulin delivery based on CGM readings, improving blood glucose management. Personalized treatment plans and decision support tools use AI-driven applications to provide personalized recommendations for insulin dosing, food and physical activity based on individual data. AI enhances telemedicine platforms by facilitating remote monitoring of person-level data, allowing healthcare providers to make informed decisions remotely. In the future AI, using advanced predictive analytics could predict the progression of diabetes and related complications, allowing for earlier and more precise interventions. AI models could assess individual risk factors and predict the likelihood of complications such as neuropathy, retinopathy, and cardiovascular disease. AI will be able to integrate data from various sources (CGMs, insulin pumps, wearables, electronic health records) to provide comprehensive and personalized care recommendations. AI systems could make real-time adjustments to treatment plans, including medication dosages and lifestyle changes, based on continuous data analysis. AI could provide personalized education and support, helping patients manage their condition more effectively through tailored advice and motivation. Chatbots and Virtual Assistants could offer 24/7 support, answering questions and providing guidance on diabetes management. AI will help accelerate the discovery of new medications and treatment options by analyzing vast amounts of data and identifying potential therapeutic targets. AI can also help to optimize clinical trial design and subject recruitment, making the process more efficient and inclusive.

Areas of attention in the application of AI in healthcare include data privacy and security. AI systems collect and process a large amount of personal health data, and can raise concerns about data privacy and security. Good security practices such as SOC2 and HiTrust certifications can help alleviate these concerns. Accuracy and reliability must be addressed. AI algorithms are not infallible and can make errors in predictions and recommendations. AI hallucinations are incorrect or misleading results that AI models generate. These errors can be caused by a variety of factors, including insufficient training data, incorrect assumptions made by the model, or biases in the data used to train the model. Using well-represented data sets and testing models for data drift over time can help increase accuracy and reliability of models. The accuracy of AI depends on the quality of data it is trained on. Incomplete or biased data can lead to inaccurate outcomes. Needless to say, garbage in will lead to garbage out. While AI can provide valuable support, it is essential to have human oversight to interpret and validate AI-driven recommendations. Over-reliance on AI tools may lead to reduced patient autonomy in managing their own health. Partnership with the clinical team will be critical. Additionally there are important ethical considerations (bias, fairness, toxicity). AI systems can perpetuate existing biases if not carefully designed and monitored, potentially leading to disparities in care. Statistical methods and tools exist for managing such considerations. Ensuring transparency in AI decision-making processes is essential for trust and accountability.

### 5.6 Addressing the quintuple aim

Digital health should strive to improve patient outcomes, provider satisfaction, healthcare costs and quality of healthcare, along with an emergent and important theme from the IHI Quintuple Aim, with the new 5^th^ aim addressing health inequity (25). This is foundational for value and sustainability and will involve a shift in focus from a downstream, reactive, disease management approach to an upstream proactive, preventive approach. Health inequities are estimated to cost the U.S. healthcare system approximately $83 billion annually (25). With social determinants of health (SDOH) driving 70% of health outcomes (26), efficient ways to assess then address SDOH through linking to local community resources are needed and could include, for example, AI-enabled geo-location capabilities to identify local food banks for food-insecure individuals. In addition, many risk factors for disease, disease severity and disease progression including SDOH are captured in medical records by clinicians in the free text but rarely translate to what exists in the structured data. Natural Language Processing (NLP) capabilities can be used to efficiently scan this text and thus help clinicians and healthcare systems identify at-risk patients. At the same time, it is important to test digital health tools that use generative AI to assure freedom from unfairness and bias which could be unintentionally introduced during the AI model training process.

Even before the pandemic, many rural, vulnerable, and underserved populations were, for a variety of reasons, struggling to keep in-person clinic visits, but compromised care resulted because of lack of regular contact. During the pandemic, those populations have largely embraced remote interactions. In fact, there is growing evidence that digital health can be a cost-effective and powerful tool for such populations (27). Digital health provides the continuity of care required to manage chronic conditions by reaching people who might otherwise feel reluctant to seek care for a condition because of privacy and confidentiality concerns or because of the stigma still often associated with certain behavioral health conditions or social situations. Digital health can also serve as a bridge to populations that are ethnically and linguistically diverse—not only patients and their families but also their care teams.

It’s widely accepted that better management of chronic illness depends on improved collaboration across the healthcare continuum. Digital health solutions can help by creating a single source of rich and readily accessible person-generated health data. Consider, for example, that with diet as a key factor in many chronic health conditions, healthcare entities are examining ways to prescribe and get reimbursement for encouraging healthier eating, especially among underserved populations. For example, with a prescription, some regions are experimenting with providing low-income residents with food vouchers redeemable at neighborhood grocery stores, convenience stores, and farmers markets, resulting in increased intake of fresh fruits and vegetables (28). Digital health solutions have the potential to connect public health, primary care, specialty care, and community resources in order to speed and simplify those types of interventions, thus improving care and lowering costs for all.

It is recognized that physical activity, food tracking, patient distress level, and social determinants of health are also important drivers of both health and wellness. Digital solutions can bring together all of those types of data from multiple devices in order to significantly improve care. The anonymity of technology can encourage candor in the self-reporting of mental state and other factors, and the connection to a wealth of additional data can give clinicians more integrated and actionable information to enrich the healthcare interaction. That direct line of sight to the whole person is a crucial aspect to effective care.

## 6 What practical steps are needed to become a digital health-ready clinical practice?

Digital health tools will inherently affect the patient care process and will introduce some changes to clinician workflow. Burdensome and time-consuming changes will impede the use of the new technology. Based on the experience and knowledge of the authors, here are some suggestions:

### 6.1 Designate a digital champion or technology navigator in the practice or clinic

This individual may be a diabetes care and education specialist, a medical assistant, nurse, or other designated staff. This individual becomes the technology expert for the practice, developing efficient workflows for helping people with diabetes identify and get started on various diabetes technology tools (29). In addition, the role helps assure that the data generated from use of the technology is available for use at clinic visits. In a feasibility evaluation exploring the potential role of a technology navigator in an academic practice, a sample of visits pre- and post-technology navigator implementation (*n* = 173) showed a 22% (41% vs. 19%) increase in patients who successfully shared their data from home before their visit and a 52% (67% vs. 15%) increase in visits where data were available to the provider for review before the appointment, whereas billing claims for continuous glucose monitor interpretation increased by 86% during the same period. Incorporating a digital champion or navigator role may improve data availability, decrease time spent on non-billable activities, and support data interpretation and billing (30).

### 6.2 Define workflow and responsibilities for a data-informed practice model

As technology is integrated into clinical care, it will be important to determine and redesign workflows to optimize resources and integrate the data into decision-making. It will be important, for example, to define protocols for remote physiological monitoring and population health management. Determine who on the clinical team will receive, review, and respond to person and population level data. Work must be done to allow integration of the data from various connected technologies into the most commonly used electronic health records (EHR) to allow for proper documentation and quality management.

### 6.3 Integrate technology into the clinical workflow using the ADCES identify-configure-collaborate technology framework

**Figure 2:**
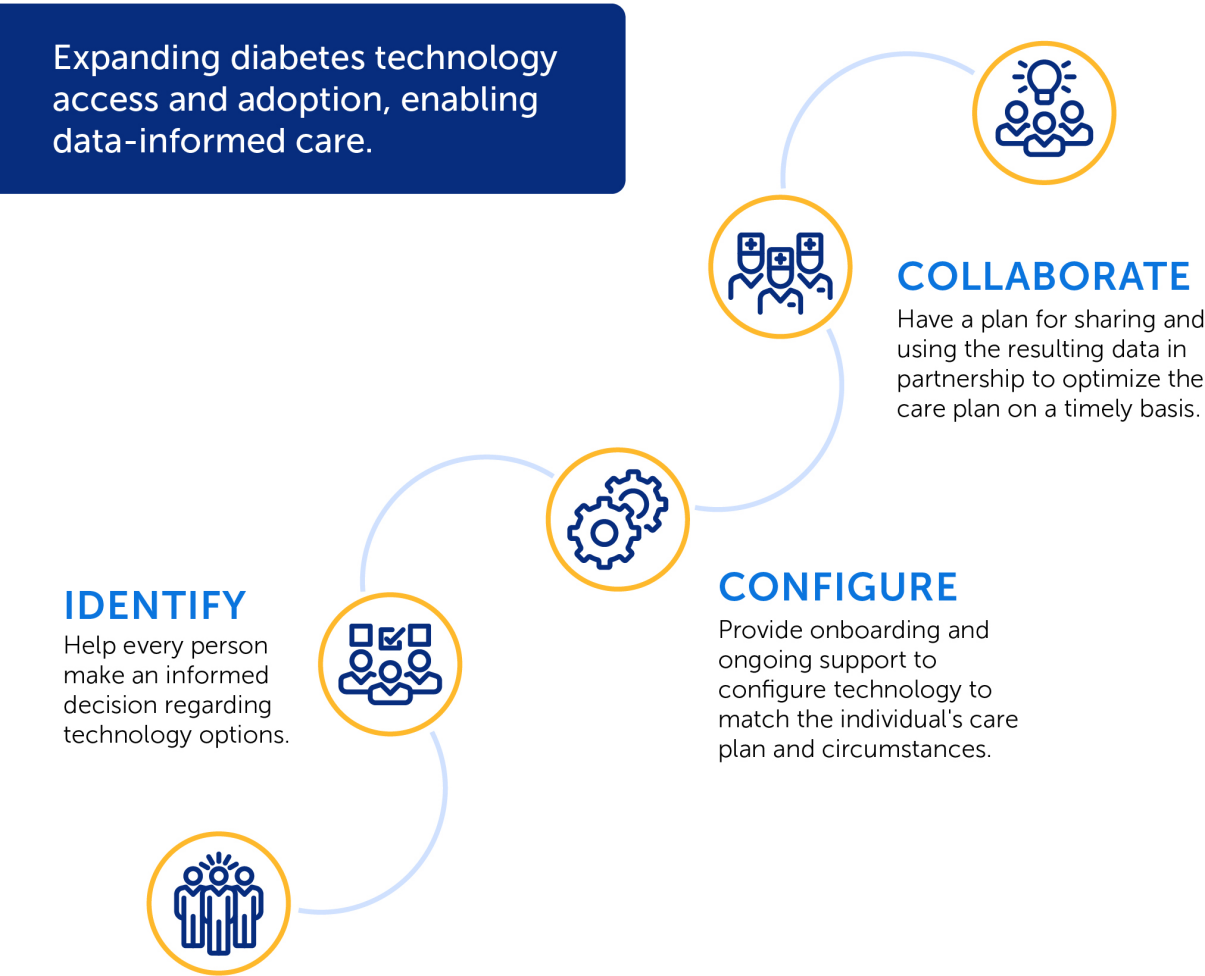
Digital Health Workflow.

This framework serves to expand technology access and adoption and enable data-informed care in a standardized way in clinical practice (31) (**Figure 2**).

#### 6.3.1 Identify

Help every person with diabetes make informed choices regarding technology as a standard of care. Recognize that choices will evolve over time.

#### 6.3.2 Configure

Provide onboarding and ongoing support and training to help individuals get off to a strong start with their technology choices, customizing the technology to match the individual’s therapy and lifestyle. The digital champion or technology navigator can serve to show individuals how to check their email on their phone, how to connect to other connected devices, and preparing individuals ahead of time to come to their onboarding or clinical appointment with their sign-in credentials for the app store or other connected technologies such as their CGM system and/or with the desired application already downloaded on their phone. Developing practice protocols for the electronic health record for the configuration steps and data output for various technologies can help standardize best practices.

#### 6.3.3 Collaborate

Develop a plan for ongoing use of both the person and population level data to assess and address barriers to following the care plan and incrementally adjusting the care plan as needed in a continuous feedback loop to disrupt therapeutic inertia.

## 7 Discussion

An increasingly supportive policy landscape, and a growing base of evidence are paving the way for digital health to become an integral part of care delivery. Nevertheless, successful integration of digital health into mainstream healthcare has been slow, complicated by a confusing landscape, worries about health data privacy, and questions about who pays for it. Evidence-based digital health solutions could spark a care delivery revolution that would dramatically improve the management of chronic illness, ease clinician burden, and lower costs. As disruptors to traditional care models, innovative healthcare organizations must meet infrastructure constraints head-on. Digital health solutions rely on powerful, reliable, and highly portable tablets, mobile devices, and laptops that give them the ability to deliver care not only inside hospital walls but also beyond including at a person’s home, which is increasingly becoming a healthcare hub. Because of that, organizations must consider infrastructure and interoperability and the ways a person’s data could move seamlessly and securely across the care continuum. Healthcare organizations will have to take the lead, but they won’t be able to do it alone. Addressing infrastructure challenges on such a scale must be a collaborative effort that includes multiple parties and entities from regulators and policy makers to health technology vendors, EHR systems, and even entities that deliver broadband—so that the reach of digital health can be extended to rural communities.

Solutions should easily and seamlessly connect to data sources and devices without added burdens on care teams or consumers. Users should be able to pair any device, such as activity trackers, blood glucose meters, blood pressure monitors, and weight scales. Users should be able to connect to labs, pharmacies, and EHRs to ensure consistency of data as well as removal of the friction associated with manual data entry. Patient data should be encrypted securely and live in the cloud so it can be accessed easily and not be lost. And patients should have full control over who can access it. When providers do access such data, they should not need a separate portal. A preferred option is a HIPAA-compliant, fully agnostic application programming interface that can work with any EHR or technology. With such a flexible and reliable infrastructure in place, clinicians could more readily combine digital solutions and other aspects of digital health—including telehealth—to create highly effective, highly efficient virtual house calls for managing chronic illness. If, for example, a clinician is managing a patient with diabetes, the clinician should, prior to a telehealth visit, be able to receive information that combines data on such health variables as the patient’s blood glucose levels, food tracking, weight, physical activity, and level of diabetes distress. That information could be paired with evidence-based, personalized clinical recommendations, thereby optimally preparing the clinician to observe self-care in the home environment before guiding a patient through next steps in their care plan.

Digital health can serve as a force multiplier to address healthcare challenges. Potential future areas of digital health advancement and research include highlighting the compelling possibilities and unresolved challenges for advancing trustworthy digital technology for the benefit of all people across society at every stage of their lives. We must identify the structural, technical, and policy preconditions for long-term progress as well as the critical priorities for cooperation and collaboration between policy makers, practitioners, and industry leaders to propel the development and application of best-in-class digital health tools. It will be important to prioritize ethical research addressing issues of user consent and addressing socioeconomic disparities in access and effectiveness. It is also important to consider the impact of digital health on health outcomes and the cost-effectiveness of service delivery. Building evidence on engagement and outcomes through strategic prospective and retrospective studies is needed. It should be recognized that digital health requires a different approach to research with the intervention continuously changing.

It is an exciting yet stressful time for healthcare. Care models such as value-based care are coming and may eventually replace traditional fee-for-service models. Younger clinicians, often coming from the “iPhone generation,” are entering the workforce and are more likely and willing to embrace technology solutions that help them and their patients. Technological solutions are improving and are incorporating more AI features. AI-powered health technologies enable true person-centered care for entire populations at scale, moving from mass generalization to mass customization. Fostering health technology literacy and confidence among those living with chronic conditions, as well as addressing the digital divide, will be essential to reach these goals. The time is ripe for implementing thoughtfully designed digital health tools that strengthen the connection between the individual and their care team and enable continuous, data-informed, on-demand diabetes care and education.

## Data Availability

The original contributions presented in the study are included in the article/supplementary material, further inquiries can be directed to the corresponding author/s.
